# Serum neurofilaments for motoneuron and dementia diseases: a German multicenter cohort study

**DOI:** 10.1007/s00415-026-13878-y

**Published:** 2026-06-02

**Authors:** Lorenzo Barba, Petra Steinacker, Steffen Halbgebauer, Patrick Oeckl, Bernhard Landwehrmeyer, Jochen Weishaupt, Federico Verde, Nicola Ticozzi, Vincenzo Silani, Johannes Levin, Sonja Schönecker, Johannes Kornhuber, Johannes Prudlo, Matthias L. Schroeter, Klaus Fassbender, Klaus Fliessbach, Janine Diehl-Schmid, Holger Jahn, Martin Lauer, Johannes Dorst, Angela Rosenbohm, Samir Abu-Rumeileh, Sarah Anderl-Straub, Marie Söntgerth, Fabiola Böhm, Jens Wiltfang, Markus Otto

**Affiliations:** 1 Department of Neurology, Martin-Luther-University of Halle-Wittenberg, Ernst-Grube-Strasse 40, 06120 Halle (Saale), Germany; 2 Department of Neurology, Ulm University Hospital, Ulm, Germany; 3 German Center for Neurodegenerative Diseases (DZNE) Ulm, Ulm, Germany; 4 Department of Neurology and Laboratory of Neuroscience, IRCCS Istituto Auxologico Italiano, Milan, Italy; 5 Department of Pathophysiology and Transplantation, Dino Ferrari” Center, Università Degli Studi Di Milano, Milan, Italy; 6 Department of Neurology, LMU University Hospital, LMU Munich, Munich, Germany; 7 Department of Psychiatry, Friedrich-Alexander University Erlangen-Nuremberg, Erlangen, Germany; 8 Department of Neurology, University of Rostock, and German Center for Neurodegenerative Diseases (DZNE), Rostock, Germany; 9 Clinic for Cognitive Neurology, University Clinic Leipzig, and Max Planck Institute for Human Cognitive and Brain Sciences, Leipzig, Germany; 10 Department of Neurology, Saarland University, Homburg, Germany; 11 Department of Neurodegenerative Diseases and Geriatric Psychiatry, University of Bonn and DZNE Bonn, Bonn, Germany; 12 Department of Psychiatry and Psychotherapy, Technical University of Munich, School of Medicine and Health, TUM University Hospital, Munich, Germany; 13 Clinical Center for Psychiatry, Psychotherapy, Psychosomatic Medicine, Geriatrics and Neurology, Kbo-Inn-Salzach-Klinikum, Wasserburg/Inn, Germany; 14 Department of Psychiatry and Psychotherapy, University Medical Center Hamburg-Eppendorf, Hamburg, Germany; 15 Department of Psychiatry and Psychotherapy, University Hospital Würzburg, Würzburg, Germany; 16 Department of Psychiatry and Psychotherapy, University Medical Center Goettingen, and DZNE, Goettingen, Germany

**Keywords:** Neurofilaments, NfL, ALS, Biomarkers, Dementia

## Abstract

**Background:**

Serum neurofilament light and heavy chains (sNfL and sNfH) have been assessed as neuronal markers for amyotrophic lateral sclerosis (ALS) and dementias. Whereas sNfL has robust literature, systematic studies on sNfH are lacking. Here, we aimed to assess the diagnostic value of sNfH in comparison to sNfL in a broad range of neurodegenerative disorders.

**Methods:**

We measured with immunoassays sNfH and sNfL in patients recruited in the multicenter German Frontotemporal Lobar Degeneration (FTLD) Consortium (n = 340) and in a single-center German cohort (n = 290). We assessed the diagnostic accuracy of serum biomarkers for ALS and dementia subtypes and their relationship with cognitive impairment.

**Results:**

sNfH and sNfL were significantly increased in ALS (n = 90) vs. controls (n = 109) and ALS mimics (n = 56, p < 0.001), with sNfL showing higher discriminative accuracy (AUC = 0.94–0.95) than sNfH (AUC = 0.87–0.88). sNfH/sNfL ratio did not improve the diagnostic performance. Both markers were elevated in patients with dementia (n = 289) vs. controls (p < 0.001). sNfL was higher in behavioral variant frontotemporal dementia (bvFTD), primary progressive aphasia (PPA) and Creutzfeldt-Jakob disease (CJD) than in Alzheimer’s disease (AD), whereas sNfH was similar in AD, PPA and bvFTD. sNfL, but not sNfH, was correlated with cognitive impairment at baseline and cognitive decline at follow-up in AD and bvFTD.

**Conclusions:**

sNfH and sNfL are elevated in motoneuron and dementia disorders. sNfH showed good discriminative accuracy for ALS, which was slightly lower than that of sNfL. sNfL, but not sNfH, showed prognostic value for assessing cognitive decline in dementia.

**Supplementary Information:**

The online version contains supplementary material available at 10.1007/s00415-026-13878-y.

## Introduction

Neurofilament proteins are entering into neurological clinical routine as diagnostic and prognostic markers for several diseases, especially for amyotrophic lateral sclerosis (ALS) [1]. Neurofilament light (NfL) and heavy chains (NfH) can be reliably measured in both cerebrospinal fluid (CSF) and blood samples, where concentrations are remarkably increased in patients with ALS compared to healthy subjects and ALS mimics [2–5]. Blood NfL showed an excellent discriminative accuracy for ALS versus differential diagnoses and slightly outperformed NfH in previous studies [4, 6]. Still, NfL concentrations in serum (sNfL) and CSF (cNfL) are increased in other neurodegenerative diseases, including frontotemporal lobar degeneration (FTLD) spectrum, Alzheimer’s disease (AD) and subgroups of patients with Parkinson disease (PD) [4, 7–9]. Given the overlap of sNfL concentrations in ALS and FTLD, serum NfH (sNfH) appeared to be more accurate for discriminating the two groups [4]. Despite preliminary data on sNfH are available in ALS, AD and FTLD [10–14], comprehensive studies comparing serum neurofilaments in neurodegenerative diseases including distinct diagnostic entities lack. Hence, to better understand the clinical utility of sNfH and sNfL, we aimed at testing their diagnostic value in a multicenter cohort of patients with a broad range of neurodegenerative disorders. We assessed the discriminative accuracy of serum neurofilaments for ALS and different dementia subtypes in comparison to CSF markers (i.e. cNfL and CSF NfH, cNfH). Moreover, given the lack of data, we conducted an exploratory analysis on sNfH and sNfL in different types of movement disorders.

## Methods

### Study population

We included in this study a total of 630 participants of which 340 recruited within the German FTLD Consortium study (www.ftld.de) [15] and 290 recruited at the Department of Neurology of Ulm University (Ulm, Germany), as previously described [4]. Participants underwent a comprehensive clinical and neuropsychological evaluation, neuroimaging (MRI) and CSF/blood sampling at baseline. Diagnoses were formulated according to internationally established diagnostic criteria for AD [16], bvFTD [17], ALS [18], Parkinson’s disease (PD) [19], sporadic Creutzfeldt-Jakob disease (CJD) [20], progressive supranuclear palsy (PSP) [21], corticobasal syndrome (CBS) [22] and primary progressive aphasia (PPA) (logopenic variant n = 29, nonfluent/agrammatic variant n = 59, semantic variant n = 41) [23]. Number of participants of each diagnostic group enrolled in the two cohorts is reported in Supp Table S1.

### Blood sample collection and analysis

CSF and blood samples were collected according to international standard procedures and were stored at -80 °C until analysis. NfH and NfL concentrations were quantified in CSF and serum with commercial immunoassays. sNfL and sNfH were measured in all patients with kits for the Ella microfluidic system (BioTechne, Minneapolis, USA). sNfL was measured at Ulm University in all cases. For sNfH, biomarker measurement in the two cohorts was performed by experienced technicians of two different laboratories (Martin-Luther-University Halle-Wittenberg for the FTLD Consortium and Ulm University for the Ulm cohort) by using the same immunoassay. To compare inter-laboratory variability of absolute sNfH concentrations, we measured sNfH in 10 serum samples in both centers. sNfH concentrations obtained in the two laboratories were highly correlated with each other (Spearman’s rho: 0.976) and of comparable range (mean ratio of concentrations measured in Halle / concentrations measured in Ulm = 0.98). Hence no inter-laboratory correction of absolute values was performed. CSF neurofilaments were measured with two different methods in the two cohorts, namely commercial Ella immunoassays in the Ulm cohort (both cNfL and cNfH) and commercial sandwich Elisa assays in the FTLD Consortium cohort (NfL kit from IBL, Hamburg, Germany; NfH kit from Biovendor, Heidelberg, Germany) according to the manufacturers’ instructions. Absolute concentrations obtained with Ella and Elisa are not directly comparable and cross-validation tests of cNfH and cNfL level measured with different methods were out of the scope of this study. Hence, we reported separately cNfH and cNfL obtained with different methods both in the figures (Supp. Figures S1-S2) and in Table 1. Finally, AD markers (amyloid-β1-42 peptide, Aβ42; phosphorylated tau protein 181, pTau181; total protein, tTau) were measured with commercial Lumipulse immunoassays (Fujirebio, Hannover, Germany). In all measurements, internal control samples were run together with test samples, with coefficient of variation not exceeding 15% and 20% for intra-plate and inter-plate variability.

**Table 1:** Cohort demographics

	Ctrls (n = 109)	ALS mimics* (n = 56)	ALS (n = 90)	AD (n = 67)	bvFTD (n = 75)	CBS (n = 28)	CJD (n = 11)	PD (n = 18)	PPA (n = 129)	PSP (n = 47)
Age at blood sampling (y) [mean (± SD)]	61.6 (± 16.4)	59.3 (± 13.6)	62.9 (± 13.2)	66.5 (± 8.4)	64.5 (± 8.5)	68.1 (± 7.0)	66.0 (± 8.2)	72.6 (± 6.0)	67.0 (± 6.7)	66.7 (± 7.4)
Male sex [n (%)]	50 (45.9)	39 (69.6)	54 (60.0)	31 (46.3)	48 (64.0)	16 (57.1)	4 (36.4)	15 (83.3)	70 (54.3)	22 (46.8)
Disease duration (y)	−	−	1 (1–2) [n = 90]	2 (1–4) [n = 45]	2 (1–4) [n = 60]	2 (1–4) [n = 24]	−	−	2 (1–4) [n = 124]	2 (1–4) [n = 42]
CDR SOB score	0 (0–0) [n = 23]	−	2 (0.5–2.5) [n = 9]	4 (3.5–7) [n = 46]	5 (3.5–7) [n = 59]	4 (2–7) [n = 21]	−	−	2.5 (1–4) [n = 107]	3 (2–7) [n = 38]
FTLD CDR score	0 (0–0) [n = 26]	−	2.5 (0,5–3.5) [n = 9]	5.5 (3.5–8) [n = 46]	6,5 (5–9.5) [n = 58]	6 (2.5–8.5) [n = 21]	−	−	4.5 (3–7) [n = 107]	4 (2.5–8) [n = 37]
MMSE score	29 (29–30) [n = 32]	−	27 (25–28) [n = 13]	24 (20–26) [n = 47]	26 (21–28) [n = 62]	25 (19–28) [n = 25]	−	−	24 (17–27) [n = 116]	26 (24–28) [n = 43]
Serum markers										
NfH (pg/ml)	149 (91–441) [n = 109]	163 (83–346) [n = 56]	1462 (662–2587) [n = 90]	287 (133–616) [n = 67]	295 (133–684) [n = 75]	587 (374–915) [n = 28]	2161 (1399–2621) [n = 11]	362 (236–747) [n = 18]	353 (164–814) [n = 129]	458 (254–1176) [n = 47]
NfL (pg/ml)	20.3 (14.5–29.2) [n = 109]	20.7 (14.0–29.6) [n = 56]	94.2 (63.5–148.0) [n = 89]	30.4 (24.0–39.4) [n = 67]	44.6 (28.8–61.7) [n = 73]	43.6 (32.3–62.2) [n = 28]	155.0 (95.8–259.5) [n = 11]	41.5 (33.2–54.7) [n = 18]	48.8 (33.8–77.8) [n = 128]	51.8 (31.8–66.9) [n = 47]
NfH/NfL ratio	9.4 (4.9–20.0) [n = 109]	7.3 (5.2–12.7) [n = 56]	14.7 (8.9–21.9) [n = 89]	9.6 (4.4–20.5) [n = 67]	5.7 (3–16.7) [n = 73]	13.7 (9.9–23.8) [n = 28]	12.9 (8.5–18.3) [n = 11]	10.3 (3.8–18.7) [n = 18]	7.6 (3.9–16.9) [n = 128]	11.1 (6.1–22.9) [n = 47]
CSF markers										
NfH (pg/ml)**	816 (543–1141) [n = 67] / 532.6 [n = 1]	953 (713–1302) [n = 54] / -	5326 (3669–6931) [n = 30] / 3239 (1499–4074) [n = 11]	1360 (1049–1716) [n = 19] / 323 (137–545) [n = 24]	1040 (839–1746) [n = 13] / 309 (234–706) [n = 41]	2410 (2165–2864) [n = 3] / 497 (259–704) [n = 14]	6005 (4286–9365) [n = 11] / -	1534 (1340–1785) [n = 14] / -	1301 (1011–1933) [n = 13] / 485 (242–651) [n = 74]	2195 (1626–2725) [n = 3] / 503 (360–845) [n = 28]
NfL (pg/ml)**	626 (441–962) [n = 74] / 1119 [n = 1]	593 (463–890) [n = 54] / -	4827 (2960–7152) [n = 39] / 6543 (4462–10,447) [n = 11]	1311 (981–1915) [n = 42] / 1373 (977–1721) [n = 21]	1952 (1155–3687) [n = 49] / 2285 (1103–3106) [n = 31]	2121 (1321–3201) [n = 16] / 1962 (1250–2603) [n = 12]	6116 (3517–9615) [n = 11] / -	1222 (1041–1713) [n = 16] / -	2355 (1530–3392) [n = 82] / 2644 (1620–3640) [n = 60]	1837 (1549–2931) [n = 31] / 2295 (1428–3431) [n = 22]
NfH/NfL ratio	1.35 (1.16–1.68) [n = 67] / 0.48 [n = 1]	1.48 (1.22–1.70) [n = 54] / -	1.12 (1.03–1.31) [n = 28] / 0.34 (0.27–0.37) [n = 11]	0.99 (0.85–1.12) [n = 19] / 0.21 (0.13–0.39) [n = 21]	0.38 (0.30–0.93) [n = 13] / 0.24 (0.12–0.30) [n = 31]	1.05 (0.90–1.25) [n = 3] / 0.25 (0.19–0.33) [n = 12]	1.22 (0.88–1.46) [n = 11] / -	1.33 (1.02–1.48) [n = 14] / -	0.86 (0.63–1.06) [n = 13] / 0.21 (0.13–0.26) [n = 60]	0.77 (0.71–0.81) [n = 3] / 0.30 (0.23–0.38) [n = 22]
Aβ42 (pg/ml)	−	−	1051 (801–1261) [n = 9]	522 (423–629) [n = 20]	743 (534–1037) [n = 36]	801 (680–951) [n = 14]	−	−	811 (563–1045) [n = 69]	758 (655–983) [n = 27]
pTau181 (pg/ml)	−	−	61 (40–67) [n = 9]	65.0 (48.8–80.3) [n = 20]	45.0 (35.3–62.5) [n = 36]	47.5 (35.0–63.5) [n = 14]	−	−	46.5 (35.0–61.0) [n = 69]	33 (23–48) [n = 27]
tTau (pg/ml)	−	−	401 (288–484) [n = 9]	508 (318–612) [n = 20]	324 (216–448) [n = 36]	322 (340–511) [n = 14]	−	−	377 (272–537) [n = 69]	274 (184–333) [n = 27]

*Aβ42* amyloid-beta 1–42; *AD* Alzheimer’s disease; *ALS* amyotrophic lateral sclerosis; *bvFTD* behavioral variant frontotemporal dementia; *CBS* corticobasal syndrome; *CDR* Clinical Dementia Rating scale; *CJD* Creutzfeldt-Jakob disease; *CSF* cerebrospinal fluid; *Ctrls* controls; *MMSE* Mini-Mental State Examination; *NfH* neurofilament heavy chain; *NfL* neurofilament light chain; *PD* Parkinson disease; *PPA* primary progressive aphasia; *PSP* progressive supranuclear palsy; *pTau181* phosphorylated tau 181; *SOB* sum of boxes; *tTau* total tau protein.

^*^Patients included in this group were subjects referred to the Department of Neurology of Ulm University (Ulm, Germany) with initial suspicion of motoneuron disease and which received an other diagnosis than ALS after completion of the diagnostic workup. Final diagnoses were: polyneuropathy (n = 15), benign fasciculations (n = 6), brachial plexus lesions (n = 3), hereditary spastic paraplegy (n = 2), inclusion body myositis (n = 5), PSP (n = 1), post-polio syndrome (n = 1), myositis (n = 1), myelitis (n = 1), motoneuritis complex (n = 1), fibromyalgia (n = 1), episodic ataxia (n = 1), cervical and lumbar disk herniation (n = 1), L5 radiculopathy (n = 1), multiple sclerosis (n = 1), somatic symptom disorder (n = 2), cervical disk prolaps (n = 2), spinomuscular atrophy (n = 2), multifocal motor neuropathy (n = 2), other myopathies (n = 7). Further details are available in Halbgebauer et al. (ref. 4).

^**^Data on CSF neurofilaments are reported separately for patients from the Ulm cohort and from FTLD Consortium, given that they were measured with different methods (Ella kits and sandwitch Elisa, respectively, see Methods). Numerical data from the FTLD Consortium are reported after those of the Ulm cohort.

### Statistical analysis

Statistical analysis was performed with R studio v.4.2.2 (R foundation, Vienna, Austria) and GraphPad v.8 (GraphPad Software, La Jolla, California, USA). Continuous variables were compared with Mann–Whitney *U* test and Kruskal–Wallis (followed by Dunn-Bonferroni’s post-hoc test) for two or more groups, respectively. Distribution of categorical variables was compared with chi-squared test. Adjusted analysis was performed with multivariable generalized linear regression models (GLMs) accounting for age given the association between neurofilament levels and older age [1, 24]. Sex-adjustment was not performed to increase analysis sensitivity given the smaller effect of sex differences in biomarker concentrations [24]. Correlations between continuous variables were computed with Spearman’s rho coefficient. Receiver operating characteristic (ROC) analysis was used to assess diagnostic accuracy, and best cutoff values were calculated by maximizing the Youden index. Comparison between AUC values was performed with DeLong test. Missing values were not imputed. A p-value < 0.05 was considered statistically significant.

### Study protocol approval

This study was conducted according to the Declaration of Helsinki and its recent modifications. The study protocol was approved by the local Ethics Committee of recruiting centers (Ulm 20/10 and Goettingen 100,305 for the Ulm cohort; see in www.ftld.de for the FTLD Consortium) and all participants or their legal representatives signed informed consent.

## Results

### Description of the study cohort

Demographic, clinical and biochemical data of the study population is reported in detail in Table 1. At group comparison, we found a significant difference in age (Kruskal–Wallis p < 0.001) between AD and ALS mimics (p = 0.035), FTLD and ALS mimics (p = 0.007), LBD and ALS mimics (p < 0.001), PD and controls (p = 0.008), PD and ALS (p = 0.012) as well as a significant difference in sex distribution (p = 0.004) (Table 1).

### Neurofilament protein levels in ALS and bvFTD

**Fig. 1:**
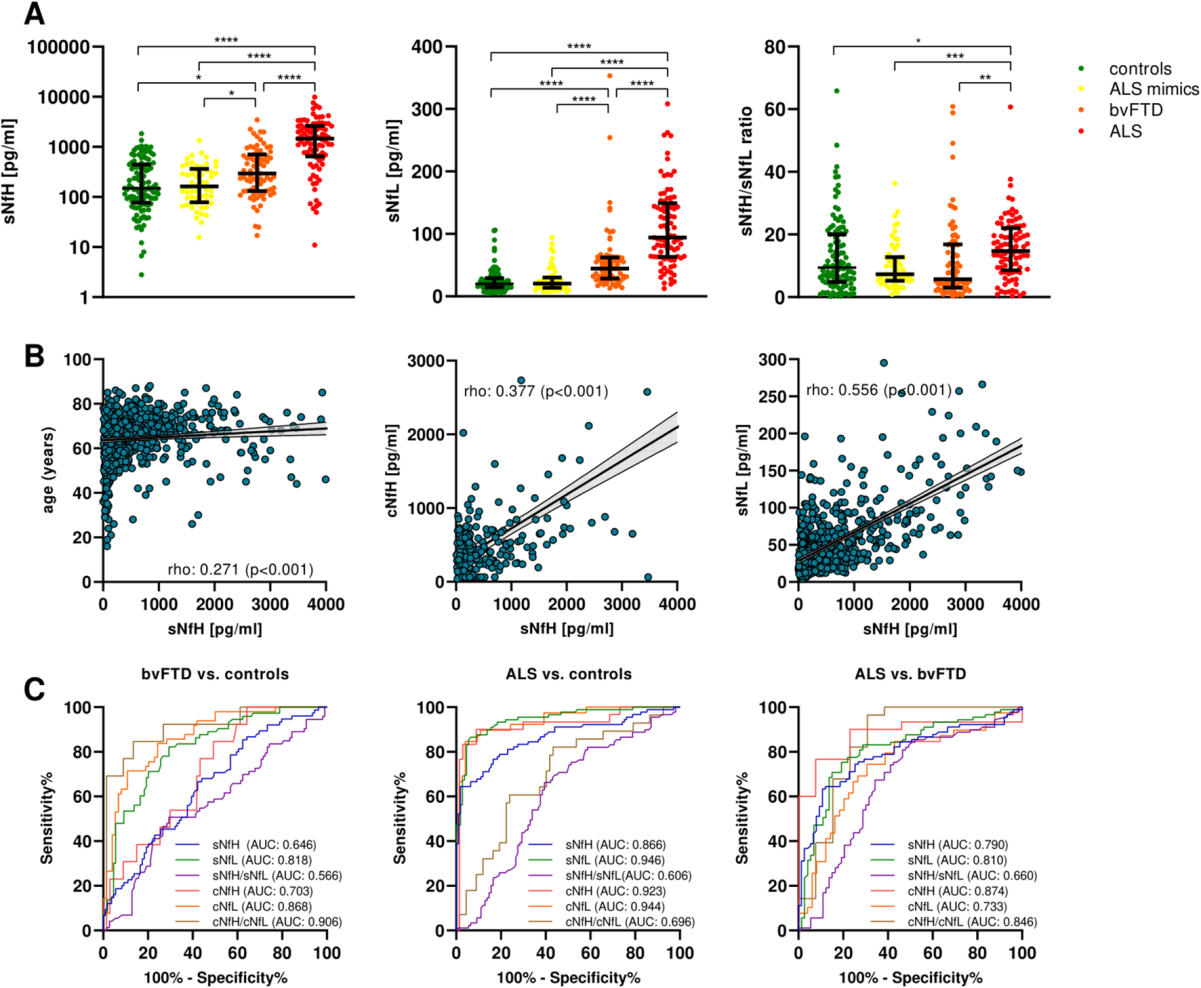
Serum neurofilament proteins in ALS and bvFTD. **A** Serum neurofilament heavy (sNfH) and light chain (sNfL) in patients with ALS, ALS mimics, bvFTD and controls. Graphs show median value and interquartile range. **B** Spearman correlations between sNfH and age, CSF NfH and sNfL. **C** Results of ROC analysis for the discrimination of bvFTD vs. controls, ALS vs. controls and ALS vs. bvFTD. *ALS* amyotrophic lateral sclerosis; *AUC* area under the curve; *bvFTD* behavioral variant frontotemporal dementia; *ROC* receiver operating characteristic analysis

Serum levels of NfH and NfL were significantly elevated in ALS [median sNfH: 1462 pg/ml (interquartile range, IQR: 662–2587 pg/ml); sNfL: 94.2 pg/ml (IQR: 63.5–148.0 pg/ml)] and bvFTD [sNfH: 295 pg/ml (IQR: 133–684 pg/ml); sNfL: 44.6 pg/ml (IQR: 28.8–61.7 pg/ml)] compared to control subjects [sNfH: 149 pg/ml (IQR: 91–441 pg/ml); sNfL: 20.3 pg/ml (IQR: 14.5–29.2 pg/ml)] and ALS mimics [sNfH: 163 pg/ml (83–346 pg/ml); sNfL: 20.7 pg/ml (14.0–29.6 pg/ml)] (Fig. 1A). The increase of sNfH and sNfL in ALS compared to controls, ALS mimics and bvFTD maintained statistical significance after accounting for age (p < 0.001 for all comparisons). The neurofilament heavy/light chain ratio (i.e., NfH/NfL ratio) in serum was significantly higher in ALS compared to control subjects (p = 0.010), ALS mimics (p < 0.001) and bvFTD (p = 0.008) at unadjusted analysis; after accounting for age and Dunn-Bonferroni’s correction, only the comparison between ALS and ALS mimics maintained statistical significance (adjusted p = 0.012) (Fig. 1).

### Correlations between neurofilament levels in serum and CSF

sNfH concentrations were weakly correlated with age in the whole cohort (rho = 0.271, p < 0.001) but not in ALS and bvFTD subgroups (results of correlation analysis in Supp. Table S2). CSF and serum concentrations of NfH were well correlated in the whole cohort with each other (rho = 0.681, p < 0.001), and sNfH level was moderately correlated with sNfL (rho = 0.556, p < 0.001) and cNfL measured with Ella (rho = 0.513, p < 0.001) (Fig. 1B) or Elisa (rho = 0.365, p < 0.001) but not with CSF AD markers (Supp Table. S2). These results were mainly driven by the ALS group, whereas no significant correlations were found in bvFTD and in AD (Supp. Table S2). Similarly, we did not find significant correlations between sNfH and AD markers in ALS and bvFTD (Supp. Table S2).

### Diagnostic performance of serum NfH and NfL for ALS and bvFTD

ROC analysis revealed a moderate accuracy of sNfH for the differentiation between bvFTD and controls [AUC: 0.646 (95%CI: 0.566–0.725)], which was significantly lower than sNfL [AUC: 0.818 (95%CI: 0.756–0.880), DeLong p < 0.001] and not significantly different from cNfH [AUC: 0.703 (95%CI: 0.567–0.840), DeLong p = 0.249] (complete results of ROC analysis in Table 2) (Fig. 1C). For differentiating ALS from controls, sNfH showed an AUC of 0.866 (95%CI: 0.813–0.920) with best cutoff (by maximizing the Youden index) of 617 pg/ml, which had a sensitivity of 76.6% (95%CI: 66.9–92.1%) and a specificity of 83.5% (95%CI: 75.4–89.3%) (Table 2). In comparison, sNfL showed a greater accuracy [AUC: 0.946 (95%CI: 0.915–0.977), DeLong p < 0.001). Ella cNfH level had a nominally higher but statistically not significantly different accuracy [AUC: 0.923 (95%CI: 0.853–0.994), DeLong p = 0.193] (for Elisa cNfH, only 1 control subject had available data, hence analysis was not performed). Similar results were observed for the differential diagnosis between ALS and ALS mimics, with an AUC of 0.880 (95%CI: 0.824–0.936), sensitivity of 75.6% (95%CI: 65.8–83.3%) and specificity of 94.6% (95%CI: 85.4–98.5%) and a best cutoff of 642 pg/ml for sNfH (Table 2). The accuracy of sNfL was higher [AUC: 0.937 (95%CI: 0.899–0.975), DeLong p = 0.035] and that of cNfH similar [AUC: 0.923 (95%CI: 0.843–1.00), DeLong p = 0.251]. Instead, the accuracy of sNfH, sNfL, cNfH and cNfL for the discrimination between ALS and bvFTD was moderate to good and not different from each other (AUC 0.733–0.914). Finally, the Nf ratio in serum was higher in ALS vs. other groups (Fig. 1A), whereas it was reduced in CSF samples of bvFTD patients (Supp. Figures S1 and S2). The discriminative accuracy of Nf ratio was not significantly better than NfL and NfH individually (Fig. 1C, Table 2).

**Table 2:** Diagnostic accuracy of serum biomarkers for differential diagnosis

Comparison	Best cutoff	AUC		Sensitivity		Specificity		PLR	DeLong p-value*
		value	95%CI	value%	95%CI	value%	95%CI		
bvFTD vs. controls									
sNfH	201	0.646	0.566–0.725	66.7	55.4–76.3	57.8	48.4–66.6	1.6	−
sNfL	26.0	0.818	0.756–0.880	82.2	71.9–89.3	70.6	61.5–78.4	2.8	< 0.001
sNfH/sNfL ratio	5.75	0.566	0.480–0.652	50.7	39.5–61.8	70.6	61.5–78.4	1.7	0.004
ALS vs. controls									
sNfH	617	0.866	0.813–0.920	76.7	66.9–84.2	83.5	75.4–89.3	4.6	−
sNfL	46.8	0.946	0.915–0.977	86.5	77.9–92.1	93.6	87.3–96.9	13.5	0.001
sNfH/sNfL ratio	12.0	0.606	0.527–0.686	66.3	56.0–75.3	60.6	51.2–69.2	1.7	< 0.001
ALS vs. ALS mimics									
sNfH	642	0.880	0.824–0.936	75.6	65.8–83.3	94.6	85.4–98.5	14.1	−
sNfL	48.2	0.937	0.899–0.975	85.4	76.6–91.3	89.3	78.5–95.0	8.0	0.035
sNfH/sNfL ratio	12.9	0.686	0.596–0.775	61.8	51.4–71.2	76.8	64.2–85.9	2.7	< 0.001
ALS vs. bvFTD									
sNfH	1080	0.790	0.720–0.860	63.3	53.0–72.6	89.3	80.3–94.5	5.9	−
sNfL	63.4	0.810	0.742–0.879	75.3	65.4–83.1	80.8	70.3–88.2	3.9	0.262
sNfH/sNfL ratio	7.75	0.660	0.573–0.748	80.9	71.5–87.7	53.4	42.1–64.4	1.7	< 0.001
dementia vs. controls									
sNfH	177	0.662	0.608–0.727	71.3	65.8–76.2	56.0	46.6–64.9	1.6	−
sNfL	26.0	0.824	0.776–0.871	82.9	78.1–86.8	70.6	61.5–78.4	2.8	< 0.001
sNfH/sNfL ratio	5.75	0.532	0.468–0.595	39.2	33.7–44.9	70.6	61.5–78.4	1.3	0.039

^*^DeLong p-values refer to comparison with sNfH

### Serum NfH and NfL in dementia subtypes

**Fig. 2:**
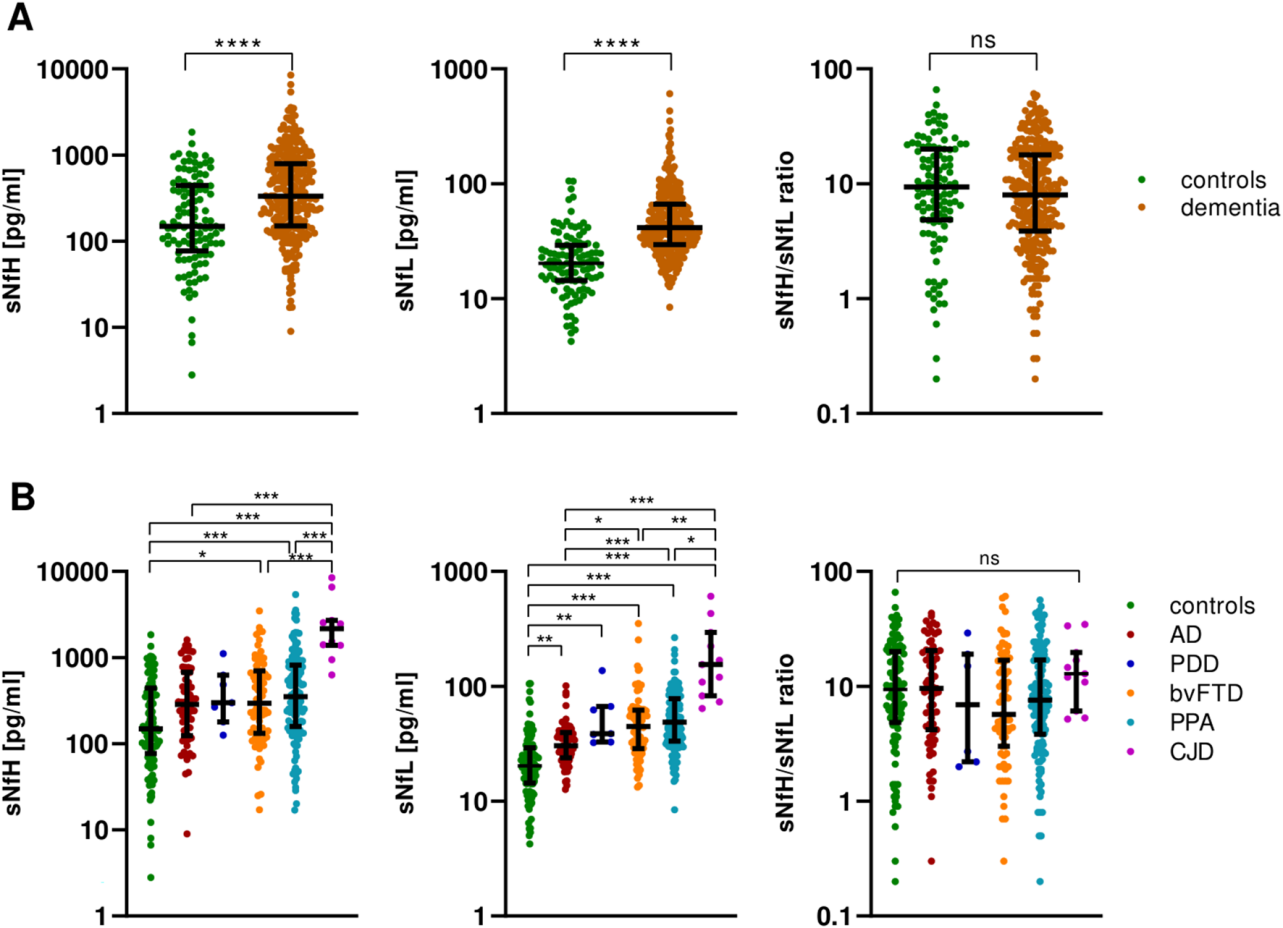
Serum neurofilament proteins in dementia subtypes. Serum neurofilament heavy (sNfH) and light chain (sNfL) (**A**) in all patients with dementia and (**B**) in dementia subgroups, namely Alzheimer's disease (AD, n = 67), Parkinson’s disease dementia (PDD, n = 7), behavioural variant frontotemporal dementia (bvFTD, n = 75), primary progressive aphasia (PPA, n = 129) and Creutzfeldt-Jakob disease (CJD, n = 11). *p < 0.05, **p < 0.01, p < 0.001

sNfH concentrations were significantly elevated in patients with dementia (n = 289, bvFTD n = 75, AD n = 67, PDD n = 7, PPA n = 129, CJD n = 11) vs. controls (n = 109), similarly to sNfL (p < 0.001 for both markers also after age-adjustment) (Fig. 2A). Among subgroups, serum neurofilament levels were higher in CJD than in bvFTD (sNfH p < 0.001, sNfL p = 0.002), AD-dem (sNfH p < 0.001, sNfL p < 0.001), PPA (sNfH p < 0.001, sNfL p = 0.018) and controls (sNfH p < 0.001, sNfL p < 0.001), as well as in AD-dem (sNfL p = 0.002), bvFTD (sNfH p = 0.017, sNfL p < 0.001), PPA (sNfH p < 0.001, sNfL p < 0.001) and PDD (sNfL p = 0.009) vs. controls. sNfH level was not different in AD, PDD and bvFTD (Fig. 2B). Moreover, the sNfH/sNfL ratio did not differ between patients with dementia and controls or across dementia subgroups (Fig. 2A and B). Diagnostic accuracy for patients with dementia was higher for sNfL [AUC: 0.824 (95%CI: 0.776–0.871)] than for sNfH [AUC: 0.662 (95%CI: 0.608–0.727), DeLong p < 0.001] (Table 2).

**Fig. 3:**
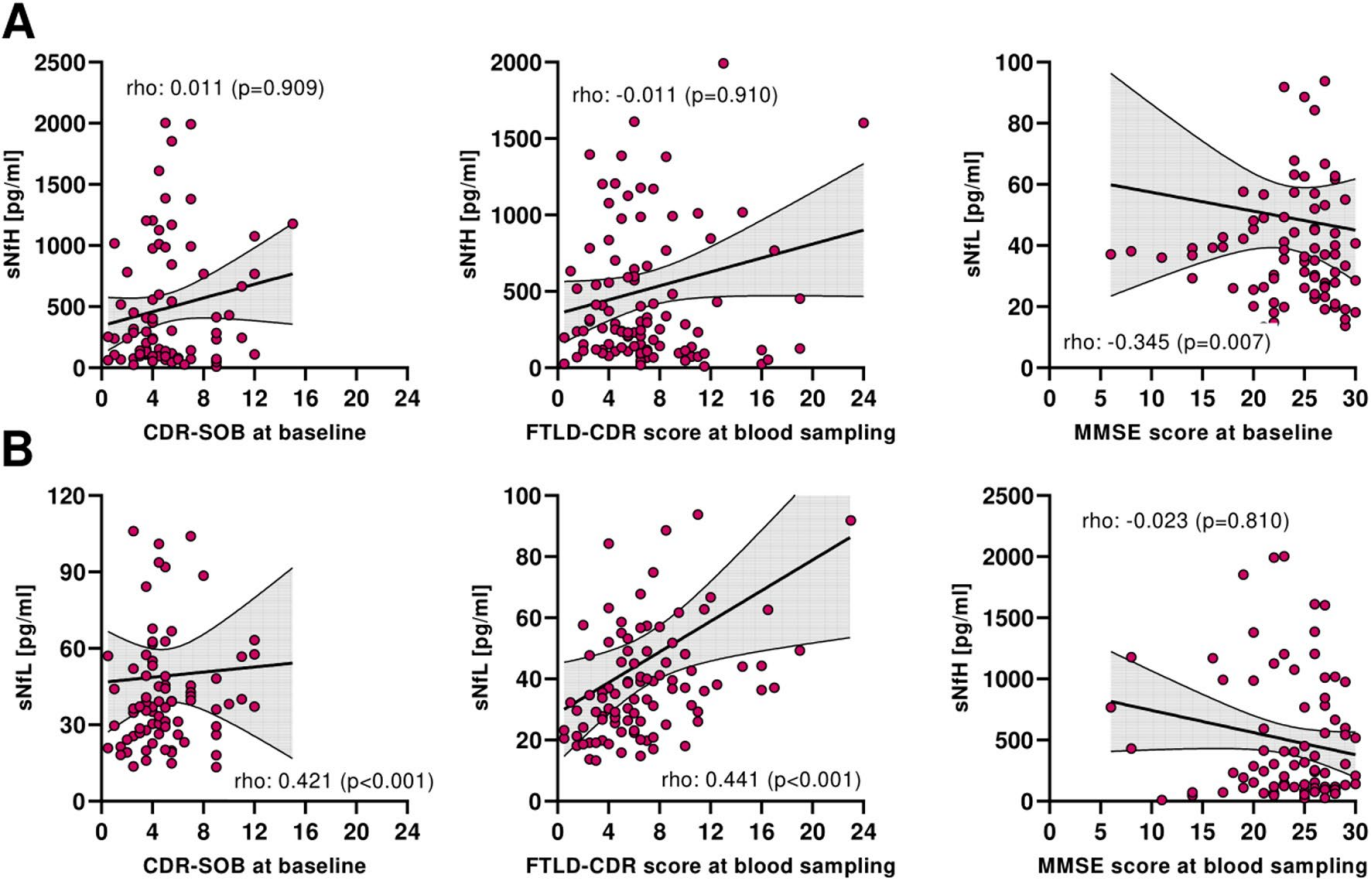
Correlation analysis between neurofilament proteins and other variables. Correlations between (**A**) serum neurofilament heavy chain (sNfH) or (**B**) serum neurofilament light chain (sNfL) and cognitive scores in patients with dementia. Correlations are reported as Spearman’s rho coefficient (p-value). *CDR-SOB* Clinical dementia rating score – sum of boxes; *FTLD* frontotemporal lobar degeneration; *MMSE* Mini-mental state examination

In an exploratory analysis, sNfH level was not significantly correlated with cognitive scores (i.e., CDR-SOB, FTLD-CDR-SOB, MMSE) evaluated at blood sampling in all patients with dementia or in bvFTD and AD subgroups (Fig. 3, Supp. Tables S3–S5). We found significant correlations between higher sNfL concentrations and cognitive scores as well as with score change at follow-up (i.e., decrease in MMSE score in points per year) (Fig. 3, Supp. Tables S3–S5).

### Serum neurofilaments in movement disorders

We explored serum neurofilament concentrations in patients with neurodegenerative movement disorders, namely PD, CBS and PSP (Table 1). We found more elevated sNfH and sNfL concentrations in patients with movement disorders compared to controls without significant difference between PD, CBS and PSP (Supp. Figure S3).

## Discussion

In this study, we investigated the diagnostic accuracy of serum neurofilament proteins (sNfH and sNfL) in a large multicenter cohort of patients and found significantly increased biomarker concentrations in motoneuron, dementia and movement disorders. Results from this study deepen our understanding of the clinical applicability of serum neurofilaments for diagnostic purposes in neurodegenerative diseases.

The high diagnostic value of sNfL for ALS has been repeatedly demonstrated in several studies [2–4, 6, 25], but the impact of several factors may limit the use of absolute concentrations in routine clinical practice. An older age, worse renal function and a lower BMI, as well as metabolic and cardiovascular comorbidities represent the most important influencing factors for sNfL [1] and are not negligible in ALS given their prevalence and clinical relevance (e.g., BMI decrease during disease course) [26, 27]. To overcome this issue, the use of age- and BMI-adjusted sNfL values and the account for renal function in multivariable analysis has been tested [28–30]. Here, sNfL confirmed also its prognostic role for predicting a more aggressive disease course and overall survival in ALS [28–30]. Still, despite the robust literature supporting the use of sNfL, other evidence suggests that sNfH could be a valid alternative marker by maintaining a clinically informative value. First, the effect of age, sex, BMI, renal function and other factors has not been confirmed yet for sNfH, which showed in another study a higher diagnostic accuracy for ALS compared to sNfL [25]. In our study, we found good diagnostic accuracy of sNfH for the differential diagnosis of ALS, confirming and expanding previous results on a smaller cohort [4]. We also provided preliminary cutoff values for Ella sNfH concentrations for discriminating ALS from controls and ALS mimics with good accuracy (AUC: 0.87–0.88), which could be further tested in independent cohorts. For this purpose, early attempts to validate adjusted sNfH values similarly to sNfL have already shown promising results [30]. Instead, both our and previous data [30] indicate that the combination of sNfH and sNfL does not carry additional clinical value to sNfL alone, also for the discrimination between ALS and bvFTD. In addition, preliminary data suggests a distinct utility of NfH and NfL as surrogate markers for assessing treatment efficacy in clinical trials for ALS. In ALS associated with *SOD1* mutations, reduction of sNfL levels as secondary outcome led to accelerated approval of the antisense oligonucleotide tofersen [31]. In observational real-world studies, NfH showed a similar strong biochemical response to treatment with tofersen already after three months of therapy when measured in CSF [32, 33], whereas longitudinal sNfH concentrations were less impacted [33]. Interestingly, sNfH decreased significantly over time after administration of the skeletal muscle activator tirasemtiv in familial or sporadic ALS, which was not observed for sNfL [34]. Hence, sNfH and sNfL should be both further tested alone or in combination for ameliorating diagnostic and prognostic models [30] and as biochemical outcome measures for disease-modifying therapies under investigation in ALS [35]. Moreover, the prognostic value of serum neurofilaments for predicting cognitive outcomes in ALS has not been explored, given the lack of systematic studies on the ALS-FTD spectrum as well as on AD copathology in ALS [36].

On another issue, serum neurofilaments were significantly increased in different dementia subtypes compared to controls. Previous studies reported higher blood NfH and NfL concentrations in AD [11] and FTD [10, 12, 13]. The main novel finding of our study is an overall elevation of sNfH in virtually all neurodegenerative dementias including AD, bvFTD, PDD, PPA and CJD, without significant differences among dementia subtypes except for higher biomarker levels in CJD. As a comparison, sNfL was found to be further increased in bvFTD and PPA compared to AD, but with limited discriminative accuracy. Hence, sNfL and sNfH may be used as surrogate markers of ongoing neurodegeneration for distinguishing patients with neurodegenerative dementias from psychiatric disorders [37]. Instead, they cannot be used alone for differential diagnosis of dementia forms given the great overlap across diagnostic subgroups, which aligns well with observations in CSF [38, 39]. As a difference between serum neurofilaments, only sNfL (but not sNfH) was significantly associated with poorer cognitive performance at baseline and with cognitive decline at follow-up. Thus, the clinical relevance, as well as the pathological correlates, of elevated sNfH concentrations in patients with dementia remains unclear. As a speculative hypothesis, higher CSF and serum NfH values in FTLD could suggest overlapping disease in the context of subclinical motoneuron degeneration with greater risk of future manifest disease, but no experimental data is available.

In a subanalysis, we explored serum neurofilaments in movement disorders and found increased sNfH and sNfL levels especially in CBS and PSP, confirming previous results [40]. Notably, the fact that we found higher levels of sNfL in PD most likely depends on the higher prevalence of cognitively impaired PD patients, which have usually greater neurodegenerative burden, and accordingly higher neurofilament levels, than PD patients without cognitive impairment [41]. Moreover, the increasing literature on the neurofilament family especially in CSF with characterization of other chains of the neurofilaments such as neurofilament medium chain (NfM) [5] raises promises for further improvement of discriminative accuracy of ALS and dementia. Methodological validation also in blood would boost investigation on the clinical value of neurofilaments in neurodegenerative diseases on the wave of NfL and NfH.

As the major limitation of our study, we acknowledge the lack of longitudinal data regarding disease progression, clinical worsening and overall survival according to the cross-sectional study design. This did not allow us to assess the prognostic or predictive value of fluid biomarkers for clinical outcomes. Also, future studies with detailed clinical follow-up data will need to unveil the influence of mixed clinical features in patients with motoneuron and dementia disease, and to assess the predictive value of sNfH and sNfL for ALS-FTD spectrum. Similarly, all patients underwent baseline MRI both as part of the diagnostic workup and of the study protocol within the FTLD Consortium [15]. However, detailed MRI data were not available for the present study, which limits understanding of the pathophysiology underlying our results. Moreover, given the promise of seed aggregation assays for TDP-43-related conditions [42] and the frequent presence of multiple pathologies in ALS and other neurodegenerative diseases [36, 41, 43], the value of serum neurofilaments in patients with evidence of multiple pathologies should be carefully evaluated. Within this frame, our dementia cohort was mainly composed from patients with bvFTD and PPA given the inclusion criteria (see Methods), whereas other dementia subgroups were less represented. Hence, the study population does not picture the frequency of dementia subtypes in real-world clinical contexts and hampered the generalizability of findings regarding differential diagnosis. This could have led us to biased results interpretation and should be taken into account in future validation studies.

In conclusion, sNfH and sNfL were significantly elevated in ALS and dementia. The diagnostic accuracy of both serum neurofilaments for ALS was high but sNfL was slightly more accurate than sNfH. sNfH and sNfL were increased in dementia but cannot be used alone for discriminating dementia forms.

## Supplementary Information

Below is the link to the electronic supplementary material.

Supplementary file1 (DOCX 25 KB)

Supplementary file2 (TIFF 88 KB)

Supplementary file3 (TIFF 115 KB)

Supplementary file4 (TIFF 171 KB)

## Data Availability

Anonymized data can be shared with qualified investigators on reasonable request to the corresponding author.
