# Supplementary material for: Serum neurofilaments for motoneuron and dementia diseases: a German multicenter cohort study
Source: J Neurol. 2026 Jun 2;273(6):356. doi: 10.1007/s00415-026-13878-y (PMC13230245; doi:10.1007/s00415-026-13878-y)
Supplement: Supplementary file 1 — Supplementary file1 (DOCX 25 KB) [file 415_2026_13878_MOESM1_ESM.docx]

**SUPPLEMENTARY FIGURES**

**Supp. Figure S1. CSF neurofilament protein in the FTLD Consortium.** Graphs show CSF levels of A) NfH, B) NfL and C) NfH/NfL ratio in patients with ALS, AD, FTLD and control subject.

**Supp. Figure S2. CSF neurofilament protein in the Ulm cohort.** Graphs show CSF levels of A) NfH, B) NfL and C) NfH/NfL ratio in patients with ALS, bvFTD, ALS mimics and control subjects.

**Supp. Figure S3.** **Serum neurofilament proteins in patients with movement disorders.** Graphs show serum levels of A) neurofilament heavy chain (sNfH), B) neurofilament light chain (sNfL), and C) neurofilament ratio (sNfH/sNfL) in patients with neurodegenerative movement disorders (MD), namely PD (including 5 cognitively unimpaired patients, 6 patients with mild cognitive impairment, and 7 patients with Parkinson disease dementia), 28 patients with corticobasal syndrome (CBS) and 47 patients with progressive supranuclear palsy (PSP).

**SUPPLEMENTARY TABLES**

**Supp. Table S1.** Number of cases per disease group in the two study cohorts.

|  |  | **FTLD consortium** | **Ulm** | **Total** |
| --- | --- | --- | --- | --- |
| Controls | healthy | 32 | 77 | 109 |
|  | ALS mimics | 0 | 56 | 56 |
| MND | ALS | 15 | 75 | 90 |
| FTLD | bvFTD | 62 | 13 | 75 |
|  | PPA | 116 | 13 | 129 |
|  | PSP | 43 | 4 | 47 |
|  | CBD | 25 | 3 | 28 |
| AD | AD-dem | 47 | 20 | 67 |
| CJD | CJD | 0 | 11 | 11 |
| LBD | PD | 0 | 3 | 3 |
|  | PD-MCI | 0 | 8 | 8 |
|  | PDD | 0 | 7 | 7 |
| **Total** |  | 340 | 290 | 630 |

**Supp. Table S2.** Spearman’s correlations of sNfH. Data are reported as Spearman’s rho coefficient (p-value) when statistically significant.

|  | **whole cohort (n=630)** | **ALS (n=90)** | **bvFTD (n=75)** | **AD (n=67)** |
| --- | --- | --- | --- | --- |
| Age at blood sampling | 0.271 (<0.001) | - | - | 0.321 (0.008) |
| cNfH | 0.681 (<0.001)  /  0.377 (<0.001) | 0.610 (<0.001)  /  - | -  /  - | -  /  - |
| sNfL | 0.556 (<0.001) | 0.717 (<0.001) | - | - |
| cNfL | 0.513 (<0.001)  /  0.365 (<0.001) | 0.695 (<0.001)  /  - | -  /  - | -  /  - |
| CSF Aβ42 | - | - | - | - |
| CSF pTau181 | - | - | - | - |
| CSF tTau | - | - | - | - |

Given that cNfH and cNfL were measured with different assays in the two cohorts (see Methods for further details), we reported separately correlations in the FTLD Consortium and in the Ulm cohort (data from Ulm reported after).

**Supp. Table S3.** Correlations between serum neurofilaments and clinical variables in patients with dementia.

|  | **sNfH [pg/ml]** | **sNfL [pg/ml]** | **sNfH/sNfL ratio** |
| --- | --- | --- | --- |
| disease duration at blood sampling (y) | 0.221 (0.023) | - | -0.250 (0.011) |
| CDR-SOB at blood sampling | - | 0.421 (<0.001) | -0.208 (0.035) |
| CDR-SOB at follow-up | -0.285 (0.026) | - | 0.199 (0.003) |
| CDR-SOB change (points/year) | - | 0.250 (0.058)* | - |
| FTLD-CDR-SOB at blood sampling | - | 0.441 (<0.001) | 0.441 (0.020) |
| FTLD-CDR-SOB at follow-up | -0.310 (0.015) | - | 0.232 (0.001) |
| FTLD-CDR-SOB change (points/year) | - | 0.254 (0.055)* | 0.254 (0.051)* |
| MMSE at blood sampling | - | -0.277 (0.004) | - |
| MMSE at follow-up | 0.284 (0.025) | -0.270 (0.035) | 0.313 (0.014) |
| MMSE change (points/year) | 0.294 (0.020) | -0.421 (0.001) | 0.382 (0.002) |

Data are reported as Spearman’s rho coefficient (p-value) when statistically significant. *Not significant at p-value <0.05.

**Supp. Table S4.** Correlations between serum neurofilaments and clinical variables in patients with bvFTD.

|  | **sNfH [pg/ml]** | **sNfL [pg/ml]** | **sNfH/sNfL ratio** |
| --- | --- | --- | --- |
| disease duration at blood sampling (y) | - | -0.268 (0.042) | - |
| CDR-SOB at blood sampling | - | 0.494 (<0.001) | -0.265 (0.046) |
| CDR-SOB at follow-up | - | - | -0.406 (0.014) |
| CDR-SOB change (points/year) | - | - | - |
| FTLD-CDR-SOB at blood sampling | - | 0.464 (<0.001) | -0.257 (0.056)* |
| FTLD-CDR-SOB at follow-up | - | - | -0.453 (0.006) |
| FTLD-CDR-SOB change (points/year) | - | - | - |
| MMSE at blood sampling | - | -0.345 (0.007) | - |
| MMSE at follow-up | - | -0.318 (0.067)* | - |
| MMSE change (points/year) | - | -0.496 (0.003) | - |

Data are reported as Spearman’s rho coefficient (p-value) when statistically significant. *Not significant at p-value <0.05.

**Supp. Table S5.** Correlations between serum neurofilaments and clinical variables in patients with AD.

|  | **sNfH [pg/ml]** | **sNfL [pg/ml]** | **sNfH/sNfL ratio** |
| --- | --- | --- | --- |
| disease duration at blood sampling (y) | 0.306 (0.041) | - | 0.335 (0.025) |
| CDR-SOB at blood sampling | - | 0.361 (0.014) | - |
| CDR-SOB at follow-up | - | - | - |
| CDR-SOB change (points/year) | -0.458 (0.028) | - | -0.433 (0.039) |
| FTLD-CDR-SOB at blood sampling | - | 0.410 (0.005) | - |
| FTLD-CDR-SOB at follow-up | - | - | - |
| FTLD-CDR-SOB change (points/year) | -0.445 (0.033) | - | -0.420 (0.046) |
| MMSE at blood sampling | - | -0.328 (0.025) | - |
| MMSE at follow-up | - | - | - |
| MMSE change (points/year) | 0.451 (0.018) | -0.382 (0.049) | 0.513 (0.006) |

Data are reported as Spearman’s rho coefficient (p-value) when statistically significant. *Not significant at p-value <0.05.
