# Supplementary figures and images for: Serum neurofilaments for motoneuron and dementia diseases: a German multicenter cohort study
Source: J Neurol. 2026 Jun 2;273(6):356. doi: 10.1007/s00415-026-13878-y (PMC13230245; doi:10.1007/s00415-026-13878-y)

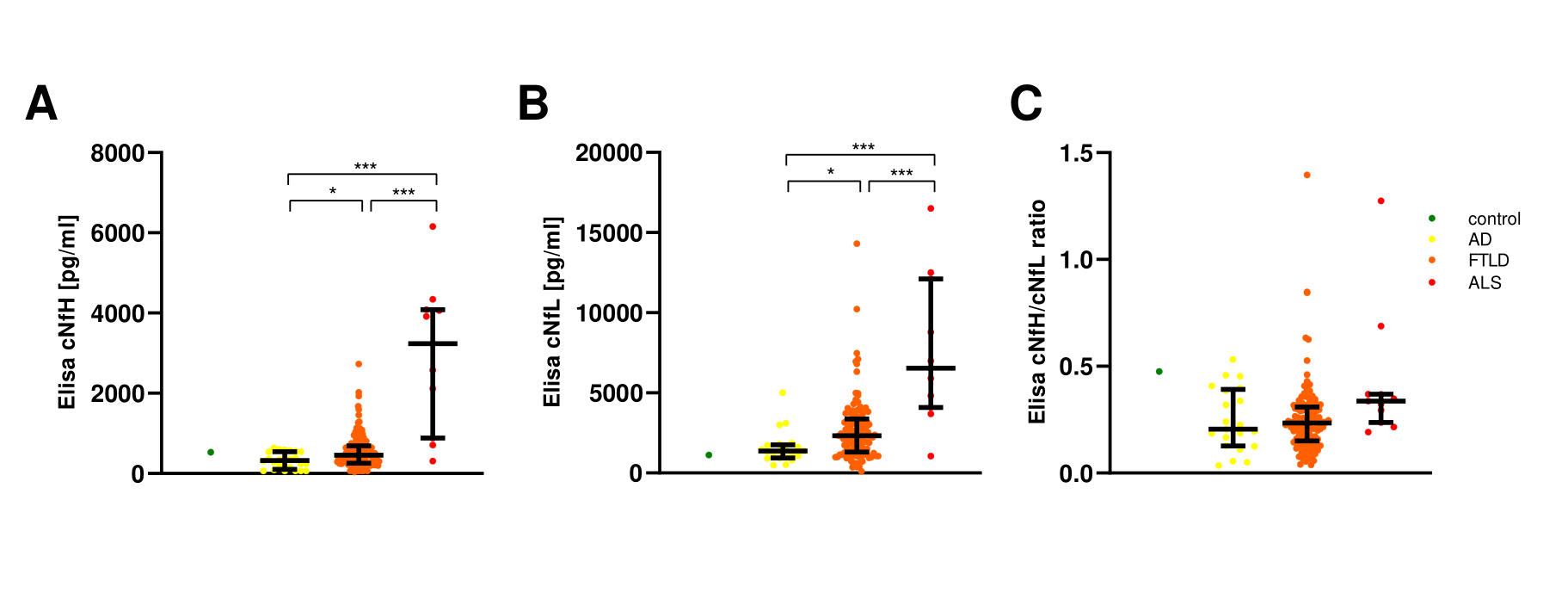

Supplement: Supplementary file 2 — Supplementary file2 (TIFF 88 KB) [file 415_2026_13878_MOESM2_ESM.tiff]

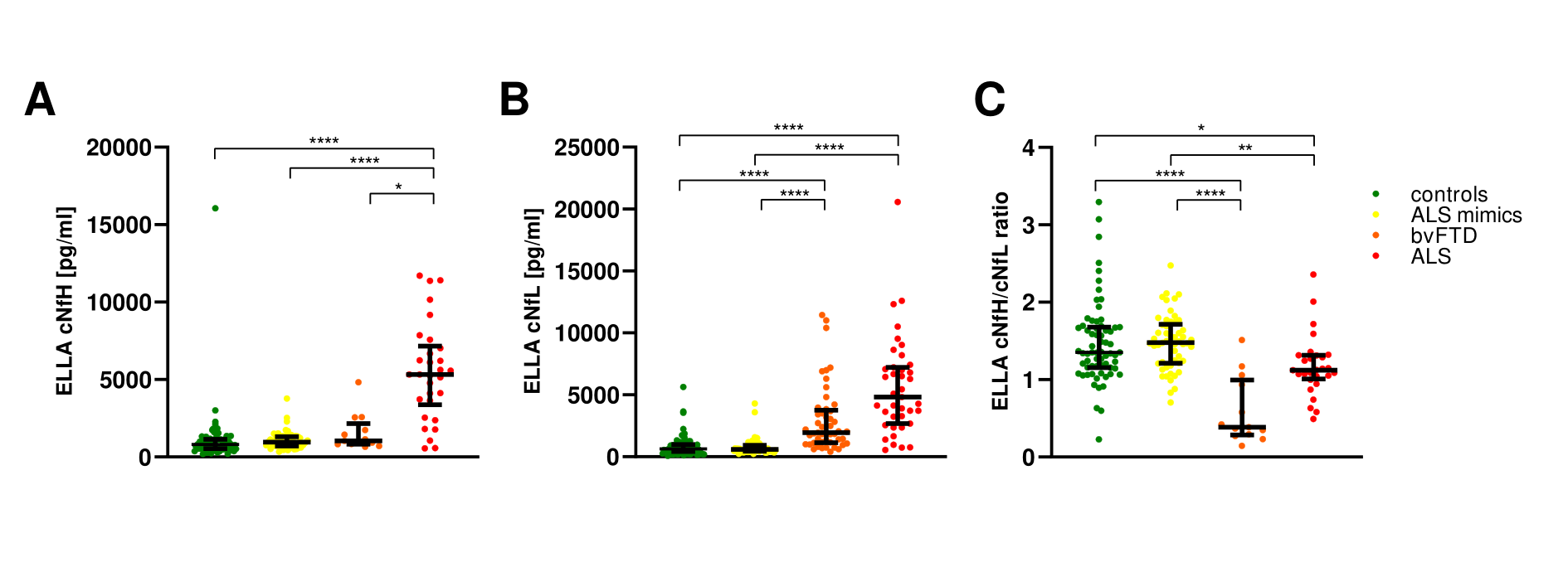

Supplement: Supplementary file 3 — Supplementary file3 (TIFF 115 KB) [file 415_2026_13878_MOESM3_ESM.tiff]

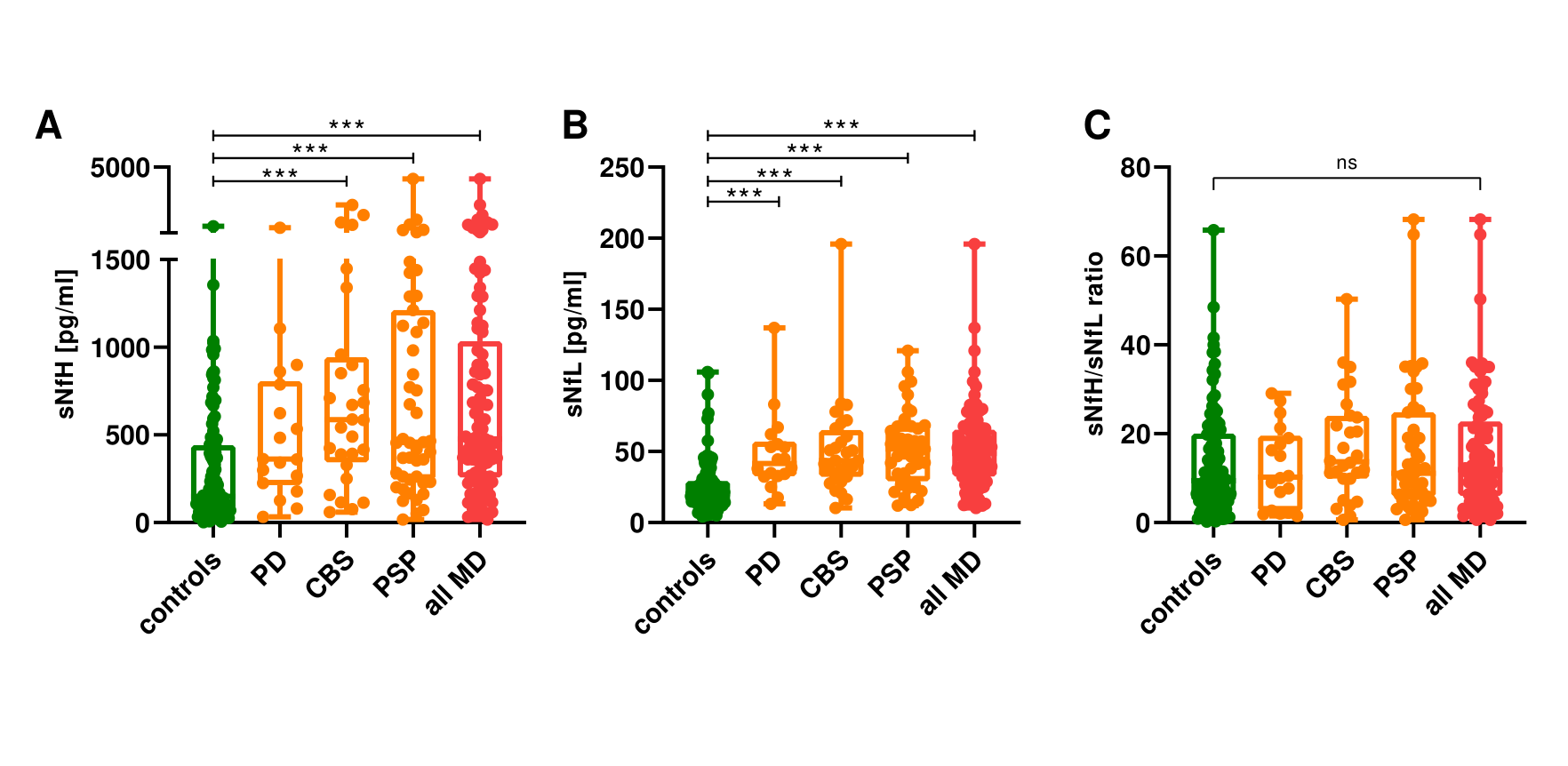

Supplement: Supplementary file 4 — Supplementary file4 (TIFF 171 KB) [file 415_2026_13878_MOESM4_ESM.tiff]
